# CCL5 derived from tumor-associated macrophages promotes prostate cancer stem cells and metastasis via activating β-catenin/STAT3 signaling

**DOI:** 10.1038/s41419-020-2435-y

**Published:** 2020-04-16

**Authors:** Renlun Huang, Shengqi Wang, Neng Wang, Yifeng Zheng, Jianfu Zhou, Bowen Yang, Xuan Wang, Juping Zhang, Lang Guo, Shusheng Wang, Zhiqiang Chen, Zhiyu Wang, Songtao Xiang

**Affiliations:** 1 The Research Centre of Integrative Cancer Medicine, Discipline of Integrated Chinese and Western Medicine, The Second Affiliated Hospital of Guangzhou University of Chinese Medicine, 510006 Guangzhou, Guangdong China; 2 Guangdong Provincial Key Laboratory of Clinical Research on Traditional Chinese Medicine Syndrome, Guangdong Provincial Academy of Chinese Medical Sciences, Guangdong Provincial Hospital of Chinese Medicine, 510006 Guangzhou, Guangdong China; 3 School of Basic Medical Sciences, Guangzhou University of Chinese Medicine, 510006 Guangzhou, Guangdong China

**Keywords:** Cancer microenvironment, Cancer stem cells, Metastasis, Prostate cancer

## Abstract

Prostate cancer stem cells (PCSCs) play a critical role in prostate cancer progression and metastasis, which remains an obstacle for successful prostate cancer treatment. Tumor-associated macrophages (TAMs) are the most abundant immune cell population within the tumor microenvironment (TME). Systematic investigation of the interaction and network signaling between PCSCs and TAMs may help in searching for the critical target to suppress PCSCs and metastasis. Herein, we demonstrated that TAMs-secreted CCL5 could significantly promote the migration, invasion, epithelial–mesenchymal transition (EMT) of prostate cancer cells as well as the self-renewal of PCSCs in vitro. QPCR screening validated *STAT3* as the most significant response gene in prostate cancer cells following CCL5 treatment. RNA-sequencing and mechanistic explorations further revealed that CCL5 could promote PCSCs self-renewal and prostate cancer metastasis via activating the β-catenin/STAT3 signaling. Notably, CCL5 knockdown in TAMs not only significantly suppressed prostate cancer xenografts growth and bone metastasis but also inhibited the self-renewal and tumorigenicity of PCSCs in vivo. Finally, clinical investigations and bioinformatic analysis suggested that high CCL5 expression was significantly correlated with high Gleason grade, poor prognosis, metastasis as well as increased PCSCs activity in prostate cancer patients. Taken together, TAMs/CCL5 could promote PCSCs self-renewal and prostate cancer metastasis via activating β-catenin/STAT3 signaling. This study provides a novel rationale for developing TAMs/CCL5 as a potential molecular target for PCSCs elimination and metastatic prostate cancer prevention.

## Introduction

Prostate cancer is the second most commonly diagnosed malignancy and the fifth leading cause of cancer death among men worldwide^1^. Prostate cancer alone accounts for approximately 13.5% of new cancer diagnoses and 6.7% of cancer-related deaths among males globally^1^. According to the International Agency for Research on Cancer, there were an estimated 1,276,106 new prostate cancer cases and 358,989 prostate cancer deaths in 2018 worldwide^1^. Prostate cancer is highly treatable in the early stage. However, most prostate cancer patients have no obvious or specific symptoms in the early stage and are usually diagnosed with distant metastases in bones and abdominal organs. The 5-year survival rate of prostate cancer patients diagnosed with local disease reached as high as 99%. However, it drops to 28% in prostate cancer patients with metastatic disease^2^. This dramatic decline in survival has driven an urgent need for discovering new strategies or targets to prevent prostate cancer metastasis.

Development of the metastatic tumor and recapitulation of the primary tumor in a secondary site is driven by cancer stem cells (CSCs)^3^. Recent evidence has also revealed the critical role of PCSCs in prostate cancer initiation and progression to metastatic disease. PCSCs possess high clonogenic, high tumor-propagating activities and are innately resistant to many therapeutics including androgen deprivation therapy (ADT), chemotherapy, and radiotherapy^4^. More importantly, PCSCs display anoikis resistance, immune evasion, tumor dormancy, and field cancerization, which contributes to prostate cancer metastasis and relapse^4^. The prognostic value of PCSCs in the clinic is very evident. PCSCs can serve as prognostic markers for overall survival in both low and high Gleason grade prostate cancers^4^. Therefore, the population size of PCSCs in the primary tumor or circulation could provide prognostic information capable of guiding prostate cancer treatment or predicting clinical outcomes. At present, several strategies have been applied for PCSCs identification, including cell surface markers (e.g., CD133, CD44, ALDH, integrin α2β1, CD117/c-kit, Trop2, c-Met, ABCG2, and so on), functional approaches (e.g., mammosphere-forming, aldefluor staining, and side-population assays) and reporter-based lineage tracing strategies^2,5^. Notably, multiple stemness-related signaling was demonstrated to be critical in controlling PCSCs self-renewal and differentiation, such as Wnt/β-catenin, Notch and Hedgehog^6,7^, etc. Many biotechnology companies and academic laboratories are currently focusing on elimination strategies that directly target PCSCs, including stem marker-based immunotherapy therapies, blocking stem signaling transduction, and modulating PCSCs metabolism^8,9^. However, targeting strategies against these biomarkers to eliminate PCSCs has been always disappointing in vivo, which might be attributed to the heterogeneous microenvironment^10^. PCSCs reside in the specific niche constituting of mesenchymal stem cells, inflammatory cells, or immune cells, which help PCSCs avoiding from killing by cytotoxic chemodrugs or immune cells. Therefore, a better understanding of PCSCs biology as well as their interaction with microenvironment components is paramount for developing novel therapeutic strategies in the clinic.

Growing evidence has indicated that targeting tumor microenvironment represents a more powerful strategy to eliminate PCSCs^10^. The PCSCs microenvironment is composed of different stromal cell populations while the most abundant cell types are cancer-associated fibroblasts (CAFs) and tumor-associated macrophages (TAMs), either resident or recruited from circulatory system^11^. It has been widely reported that CAFs exert a mandatory role in prostate cancer progression as they metabolically sustain prostate cancer cell survival and growth, recruit inflammatory and immune cells, and promote cancer cells stemness and EMT, thereby favoring metastatic dissemination^12,13^. As the most highly infiltrative immune cell population within the TME, TAMs account for 30–50% of the tumor mass^14^. Clinical analysis indicated that TAMs infiltration usually increased along with prostate cancer progression^15^, and high TAMs infiltration usually predicts poor overall survival of prostate cancer patients^16–18^, especially in the metastatic-stage prostate cancer patients^19^. For example, by analyzing a cohort containing 592 prostate cancer patients, Erlandsson et al. found that men with high TAMs infiltration in the prostate TME had increased odds ratios (OR = 1.93, 95% CI 1.23–3.03) for death^16^. While much is known about the role of TAMs in primary prostate cancer progression and prognosis, less is known about the molecular steps by which TAMs contribute to PCSCs. To our best knowledge, only Huang et al. demonstrated that the reciprocal network between TAMs and PCSCs could facilitate the stem-like properties of PCSCs, progression and androgen deprivation therapy resistance of prostate cancer^10^. Notably, the chemokines secreted by TAMs are an important messenger mediating the crosstalk between TAMs and cancer cells^20^. For example, the CC chemokine ligand 2 (CCL2, also known as MCP-1) could increase the growth and metastasis of transplanted prostate cancer xenografts in vivo^21^. Our previous study indicated that CCL5, another member of the CC chemokine family, was a more abundant TAMs-secreted chemokine than CCL2^22^. Additionally, it has been reported that the CCL5-CCR5 axis blockage in the TME may represent a potential clinically available strategy for inhibiting human breast phyllodes tumor growth^23^. Nevertheless, the prognostic value of TAMs-secreted CCL5 for prostate cancer as well as its modulatory effect on both prostate cancer cells and PCSCs cells remain unclear and deserve further investigations.

Herein, by conducting the clinicopathological sample analysis, RNA-sequencing, molecular biology experiment and animal experiment, we systematically demonstrated that CCL5 was mainly secreted by TAMs and could promote PCSCs self-renewal and metastasis via activating β-catenin/STAT3 signaling in vitro. In addition, CCL5 silencing in TAMs significantly inhibited prostate cancer xenografts growth, bone metastasis as well as PCSCs self-renewal and tumorigenicity in vivo. More importantly, elevated CCL5 expression was positively associated with high Gleason grade, poor prognosis, metastasis, and increased PCSCs activity in prostate cancer patients. Overall, this study not only revealed the underlying mechanisms by which TAMs promoted PCSCs but also uncovered TAMs/CCL5 as a novel target in predicting prostate cancer prognosis and inhibiting prostate cancer metastasis.

## Results

### TAMs-derived CCL5 was elevated in prostate cancer and associated with metastasis

**Fig. 1:**
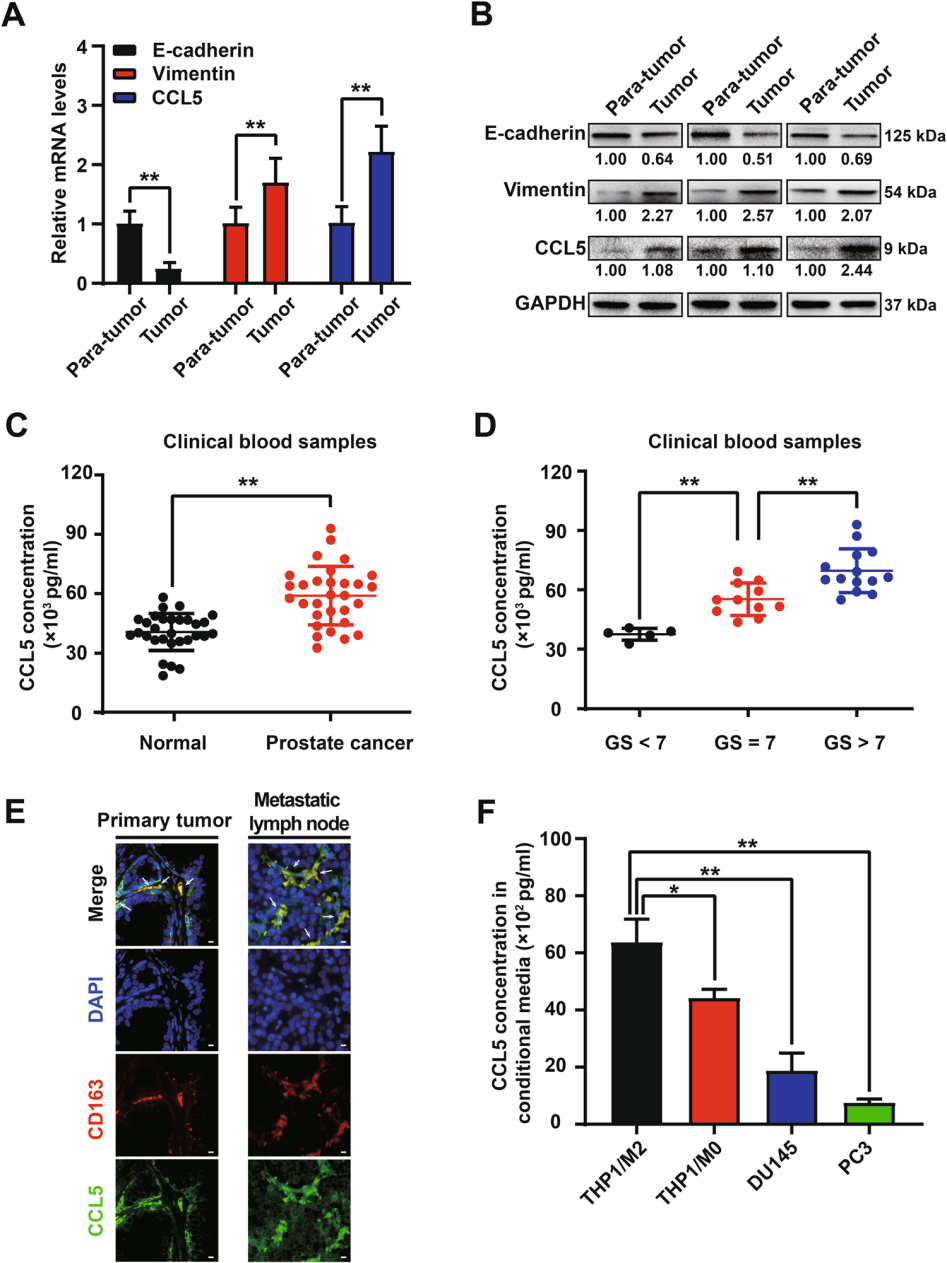
TAMs-derived CCL5 was elevated in prostate cancer and associated with metastasis. **a**, **b** The mRNA and protein expression differences of indicated genes between prostate cancer tumor tissues and para-carcinoma tissues (*n* = 3). **c** Elisa assay indicated that the blood samples of prostate cancer patients (*n* = 30) exhibited elevated expression of CCL5 when compared with that of healthy male participants (*n* = 30). **d** Prostate cancer patients with higher Gleason grade exhibited an increased CCL5 accumulation in the blood (*n* = 30). **e** Tissue immunofluorescence assay suggested that CCL5 and the macrophage marker CD163 were co-localized in both the primary tumor and metastatic lymph node of prostate cancer patients. Scale bar, 10 μm. **f** TAMs exhibited increased secretion of CCL5 than immature macrophages or prostate cancer cells. CCL5 concentrations in the cell culture supernatants of THP1-derived macrophages (M0), THP1-derived TAMs (M2), DU145 and PC3 cells were measured using Elisa method (*n* = 10). All values are presented as the mean ± SD. *p < 0.05, **p < 0.01.

Firstly, the prognostic value of CCL5 for prostate cancer patients was determined by analyzing the expression difference of CCL5 in prostate cancer specimens and nonmalignant specimens. As shown in Fig. 1a, b, increased expression levels of CCL5 mRNA and protein were observed in prostate cancer tumor tissues than para-carcinoma tissues, accompanied by the decreased expression of E-cadherin and increased expression of Vimentin. These results suggested a potential association between CCL5 accumulation and EMT of prostate cancer tissues. Elisa assay further indicated that the blood samples of prostate cancer patients exhibited elevated expression of CCL5 when compared with that of healthy male participants (Fig. 1c). High Gleason grade is a crucial indicator in predicting the advanced stage and poor prognosis of prostate cancer patients in the clinic^24^. Prostate cancer patients with higher Gleason grade exhibited an increased CCL5 accumulation in the blood (Fig. 1d). These results indicated that CCL5 accumulation might predict poor prognosis of prostate cancer patients. Our previous study reported that CCL5, also known as RANTES, was one of the most abundant chemokines secreted by TAMs^22^. Therefore, we further investigated whether TAMs were the major secretion cell type of CCL5. Tissue immunofluorescence assay suggested that CCL5 and the macrophage marker CD163 were co-localized in both the primary tumor and metastatic lymph node of prostate cancer patients^25^ (Fig. 1e). Elisa assay further convinced that TAMs exhibited a significantly increased secretion of CCL5 than the immature macrophages (M0) or prostate cancer cells (Fig. 1f). Altogether, CCL5 was mainly secreted by TAMs, and its expression was elevated in prostate cancer and associated with metastasis.

### TAMs-secreted CCL5 promotes the invasion and the PCSCs subpopulation of prostate cancer cells in vitro

**Fig. 2:**
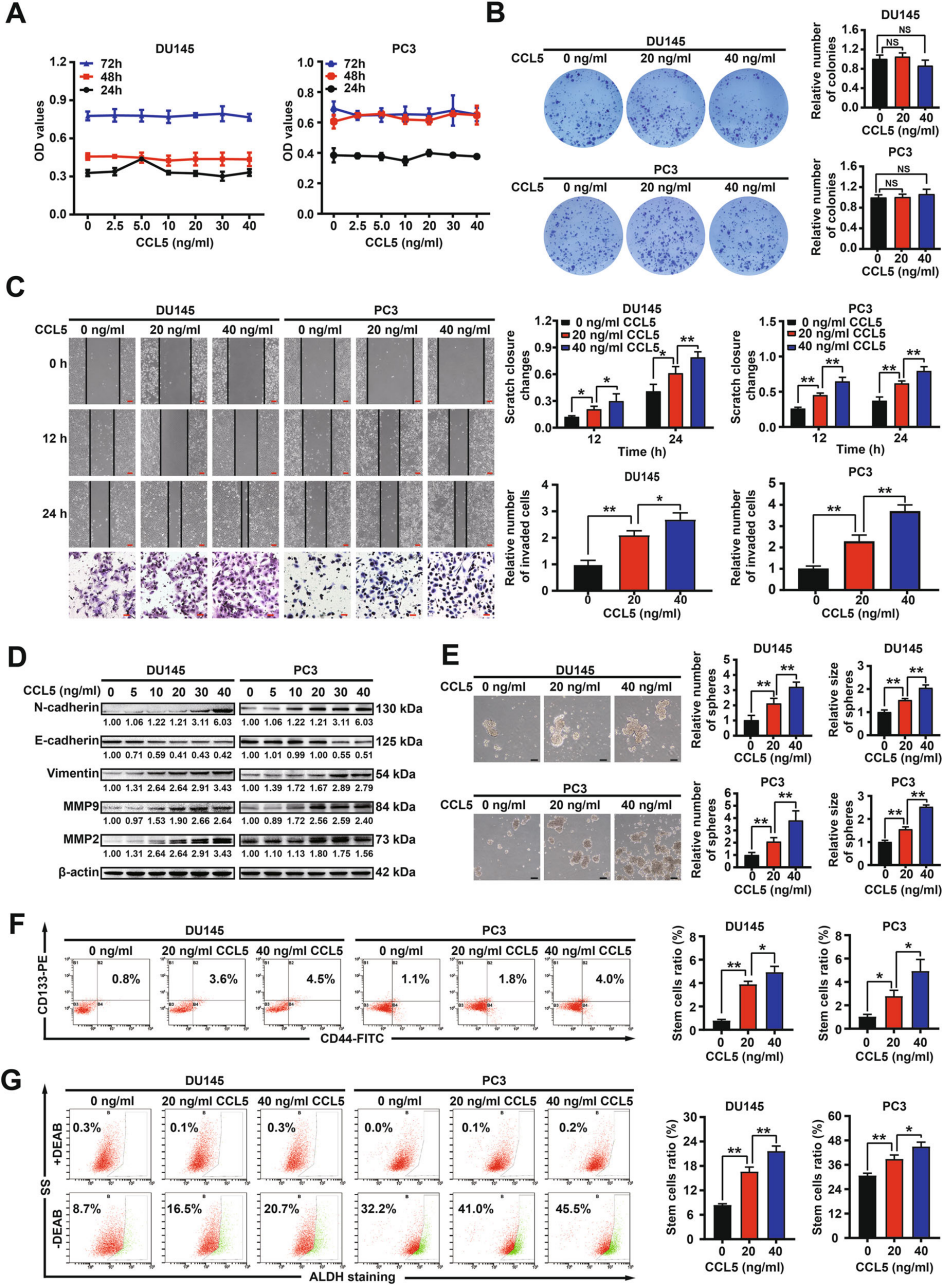
CCL5 promotes the invasion and the PCSCs subpopulation of prostate cancer cells in vitro. **a**, **b** CCL5 (2.5–40 ng/ml) had no obvious influences on the proliferation and colony formation capacities of the prostate cancer cell lines DU145 and PC3. **c** CCL5 addition (20–40 ng/ml) significantly promoted the migration and invasion of DU145 and PC3 cells. Scale bars represent 200 μm for wound healing assay images and 50 μm for transwell assay images. **d** Western blotting assay revealed that CCL5 (5–40 ng/ml) could promote EMT and metastasis of both DU145 and PC3 cells. **e** CCL5 treatment (20–40 ng/ml) significantly increased not only the number but also the size of mammospheres formed by prostate cancer cells. Scale bar, 100 μm. **f**, **g** CCL5 treatment (20–40 ng/ml) significantly elevated the CD44^+^/CD133^+^ subpopulation and the ALDH^+^ subpopulation in DU145 and PC3 cells, indicating that CCL5 could promote the self-renewal of PCSCs. All values are presented as the mean ± SD. *n* = 3, *p < 0.05, **p < 0.01.

**Fig. 3:**
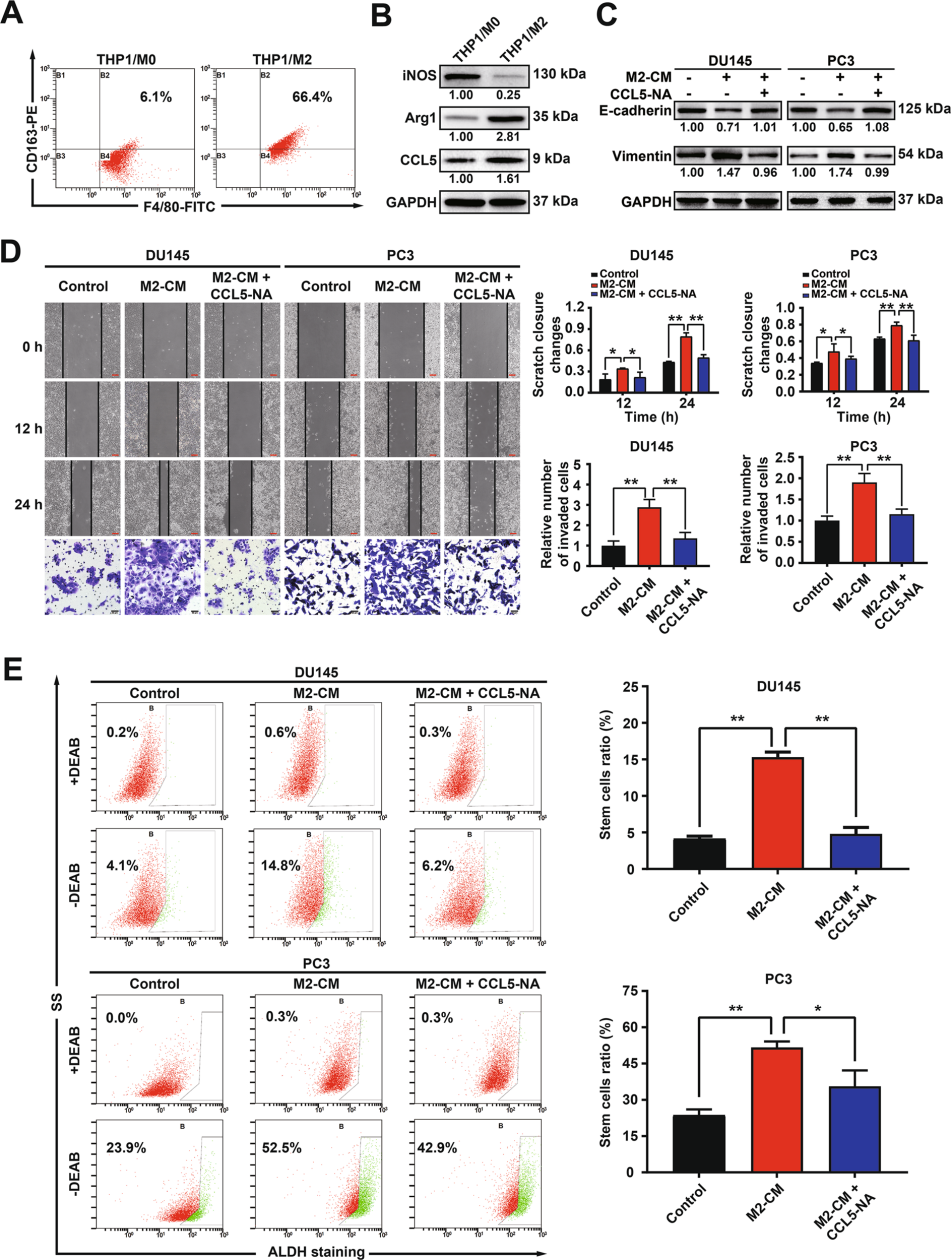
TAM-secreted CCL5 promoted the invasion of prostate cancer cells and the self-renewal of PCSCs. **a**, **b** The reliability of THP1-derived TAMs was validated by analyzing the macrophage surface markers. THP1 monocytes were induced differentiation into macrophages (M0) by 100 ng/ml PMA treatment for 24 h, which were further transformed into M2 phenotype macrophages (TAMs) by 10 ng/ml IL-4 induction for 72 h. **c**, **d** The CM of THP1-derived TAMs significantly promoted EMT, migration and invasion of prostate cancer cells, while CCL5 NA could partly abrogate that. Scale bars represent 200 μm for wound healing assay images and 50 μm for transwell assay images. **e** The CM of THP1-derived TAMs significantly promoted the self-renewal of PCSCs, while CCL5 NA could partly abrogate that. All values are presented as the mean ± SD. *n* = 3, *p < 0.05, **p < 0.01.

In order to validate the metastasis promotion effects of CCL5, we further investigated the effect of recombinant human CCL5 protein on prostate cancer metastasis using human prostate cancer cell lines DU145 and PC3. CCL5 (2.5–40 ng/ml) had no obvious influences on the proliferation and colony formation capacities of the above prostate cancer cells (Fig. 2a, b). However, CCL5 addition significantly promoted the migration and invasion of DU145 and PC3 cells (Fig. 2c). Western blotting assay further indicated that CCL5 could induce epithelial-mesenchymal transition (EMT) in both prostate cancer cells, characterized by the decreased expression of E-cadherin as well as the increased expression of N-cadherin, Vimentin, MMP2, and MMP9 (Fig. 2d). PCSCs are considered as the origin and driving force of prostate cancer progression and metastasis^26,27^. CSCs are enriched in non-adherent spherical clusters of cells, which are termed mammospheres^28^. As shown in Fig. 2e, CCL5 treatment significantly increased not only the number but also the size of mammospheres. Until now, several PCSCs populations have been identified using cell surface markers including CD44, CD133 and ALDH^2,5^. Importantly, CCL5 treatment also significantly elevated the CD44^+^/CD133^+^ subpopulation (Fig. 2f) and the ALDH^+^ subpopulation (Fig. 2g) in DU145 and PC3 cells. These findings suggested that CCL5 could promote the invasion of prostate cancer cells and the self-renewal of PCSCs in vitro. As demonstrated above, CCL5 was mainly secreted from TAMs. Therefore, we further investigated whether the conditional medium (CM) of TAMs could achieve similar effects on prostate cancer cells invasion and PCSCs self-renewal. In the present study, THP1 monocytes were induced differentiation into macrophages (M0) by PMA treatment and further transformed into M2 phenotype macrophages (TAMs) by IL-4 induction^22^. THP1-derived TAMs exhibited an increased expression of M2 phenotype markers CD163 and Arg1, accompanied by a decreased expression of M1 phenotype marker iNOS, indicating a successful induction of THP1-derived TAMs. Consistent with the Elisa assay, Western blotting assay also convinced the elevated expression of CCL5 in THP1-derived TAMs (Fig. 3a, b). Notably, the CM of THP1-derived TAMs significantly promoted the migration, invasion, EMT, and the PCSCs subpopulation of both the androgen receptor (AR)-negative prostate cancer cells (PC3 and DU145) and the AR-positive prostate cancer cells (LnCaP and VCaP), while CCL5 neutralizing antibody (NA) could partly abrogate that (Fig. 3c–e and Supplementary Fig. 1). Altogether, these results validated that TAMs-secreted CCL5 could promote the invasion and the PCSCs subpopulation of prostate cancer cells in vitro independent of AR status.

### *STAT3* is the primary gene responsive for CCL5 stimulation in prostate cancer

**Fig. 4:**
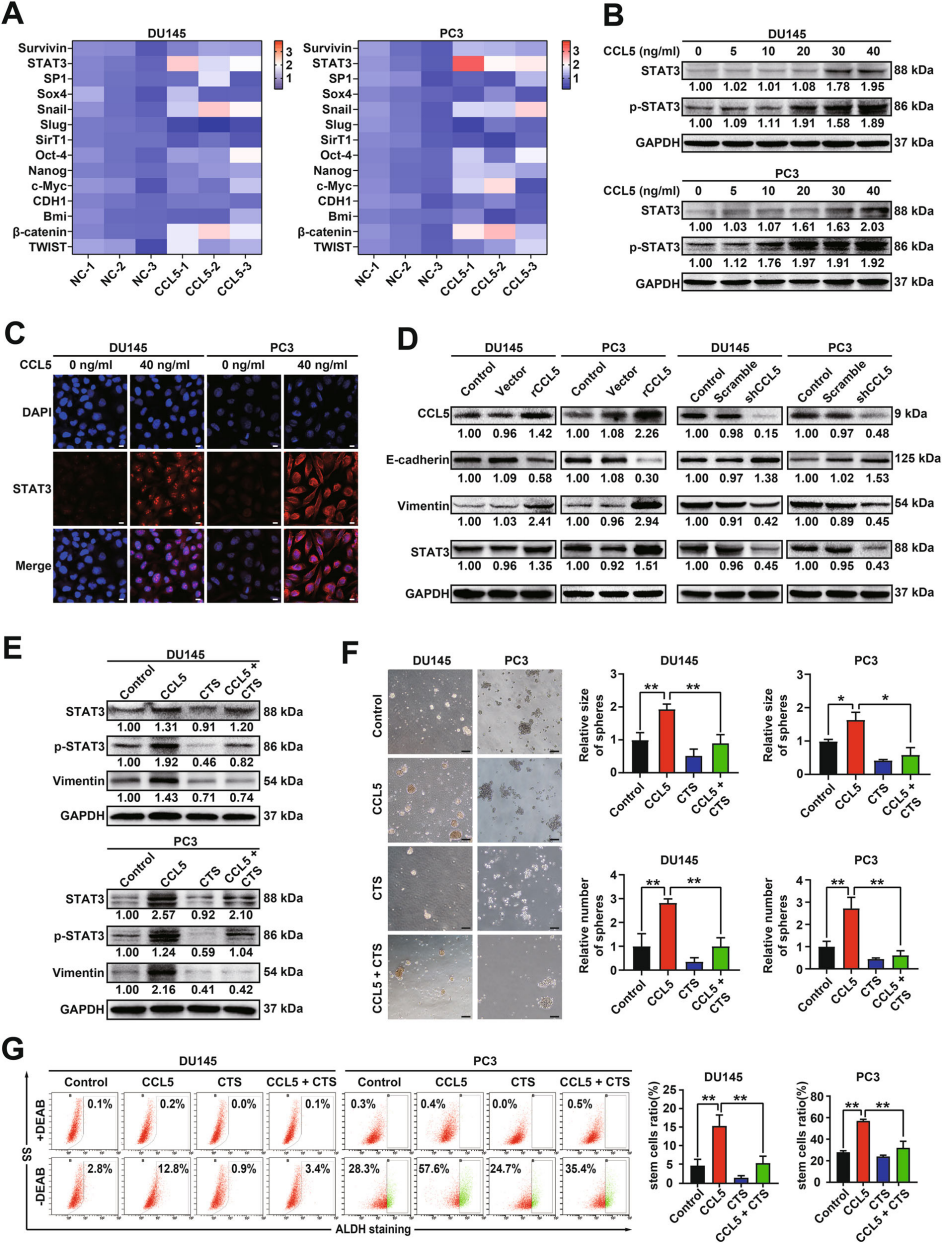
STAT3 is identified as the primary gene responsive for CCL5 stimulation on prostate cancer cells. **a** The mRNA expression differences of a panel of metastasis and stemness-related genes in both DU145 and PC3 cells after 40 ng/ml CCL5 treatment were determined by QPCR method. **b**, **c** CCL5 could significantly promote STAT3 expression, phosphorylation as well as its nuclear translocation in both DU145 and PC3 cells. Scale bar, 10 μm. **d** CCL5 overexpression significantly activated STAT3 signaling and induced EMT in DU145 and PC3 cells, while CCL5 knockdown achieved the opposite effects. **e**–**g** CCL5 treatment alone significantly activated STAT3 signaling and promoted the self-renewal efficacy of PCSCs, while cryptotanshinone (CTS), the specific inhibitor of STAT3, partly abrogated that. Scale bar, 100 μm. All data represent the means ± SD. *n* = 3, *p < 0.05, **p < 0.01.

Next, the mechanism by which CCL5 promoted the invasion and the PCSCs subpopulation of prostate cancer cells was explored. We analyzed the mRNA expression differences of a panel of metastasis and stemness-related genes in prostate cancer cells after CCL5 treatment. *STAT3* was identified as the most significant response gene among the 14 metastasis and stemness-related genes (Fig. 4a). Accumulating reports have suggested that *STAT3* is highly implicated in the development and metastasis of prostate cancer because of its extensive transcription modulatory effect on downstream genes^29^. CCL5 could significantly promote STAT3 expression, phosphorylation as well as its nuclear translocation in both DU145 and PC3 cells, indicating that CCL5 could induce persistent activation of STAT3 signaling in prostate cancer cells (Fig. 4b, c). Consistent with the effect of exogenous CCL5 addition, CCL5 overexpression by genetic approaches also significantly elevated STAT3 activity and induced EMT, while CCL5 knockdown achieved the opposite effects (Fig. 4d). To confirm the key role of STAT3 in CCL5-induced promotion effect on prostate cancer, we further investigated the combined effect of CCL5 and STAT3 inhibitor. As shown in Fig. 4e–g, CCL5 treatment alone significantly activated the STAT3 signaling and promoted the self-renewal of PCSCs, while cryptotanshinone (CTS), the specific inhibitor of STAT3, partly abrogated that. Altogether, these results validated that *STAT3* acted as the primary response gene accounting for the promotion effect of CCL5 on prostate cancer cells.

### CCL5 promotes prostate cancer invasion and PCSCs self-renewal *via* activating the CCR5/β-catenin/STAT3 pathway

**Fig. 5:**
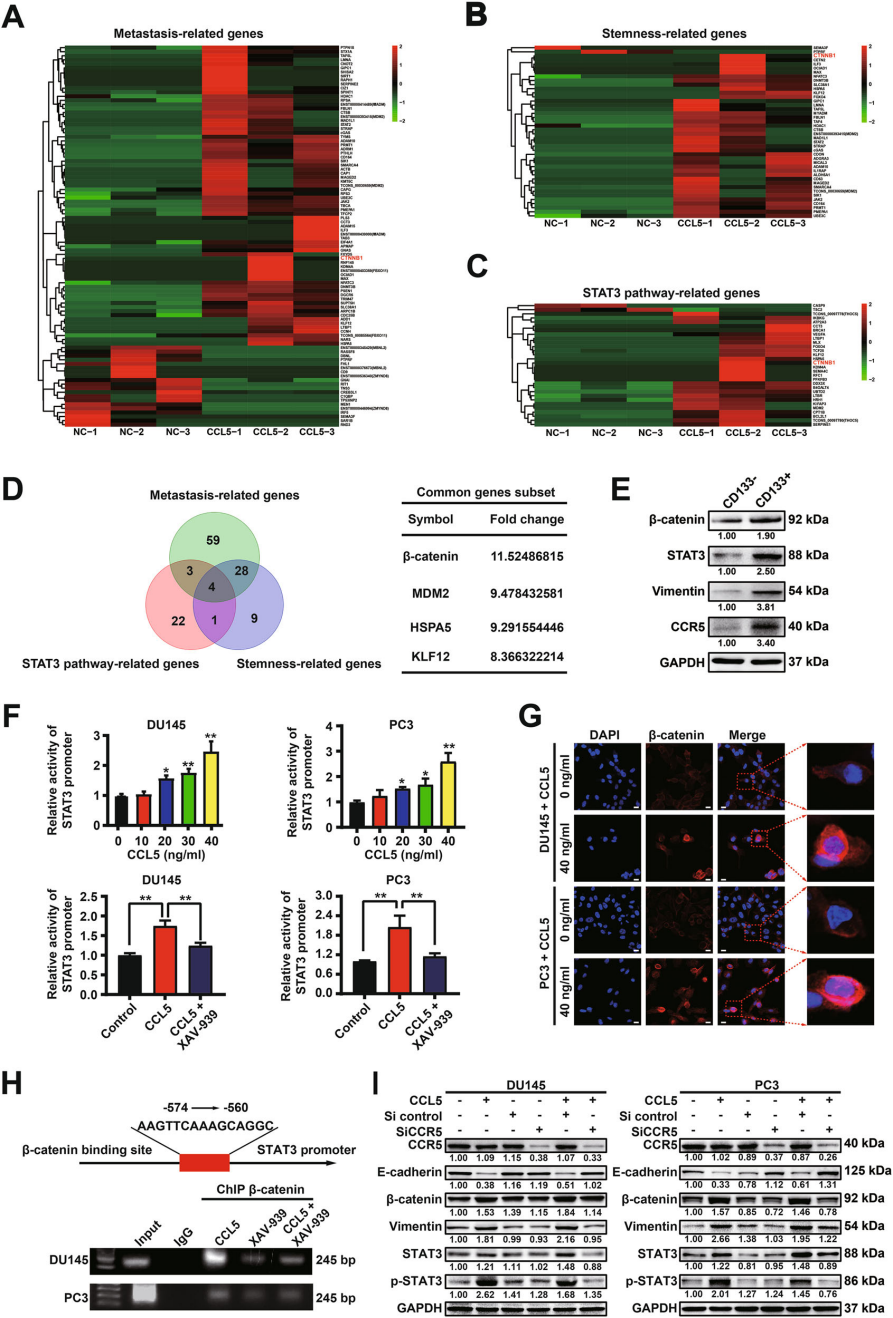
CCL5 promotes prostate cancer invasion and PCSCs self-renewal *via* activating the CCR5/β-catenin/STAT3 pathway. **a**–**c** Heatmaps of 94 metastasis-related DEGs (**a**), 42 stemness-related DEGs (**b**), as well as 30 STAT3 pathway-related DEGs (**c**). RNA-Seq analysis was conducted to characterize the cellular responses of PC3 cells to 40 ng/ml CCL5 treatment. Differential gene expression analysis was conducted to identify the DEGs (*n* = 3). **d** Venn diagram of the DEGs in the indicated groups. CTNNB1, also known as β-catenin, was the most significant one of them. **e** Western blotting assay indicated that the CD133^+^ PCSCs sorted from PC3 cells by MACS method exhibited increased expression of CCR5, β-catenin, and STAT3 when compared with the CD133^-^ subpopulation (*n* = 3). **f** CCL5 treatment significantly elevated the promoter activity of *STAT3* in PC3 cells while XAV-939, the specific inhibitor of β-catenin, partly abrogated that (*n* = 6). **g** Immunofluorescence assay indicated that CCL5 treatment (40 ng/ml) could significantly induce the expression and nuclear translocation of β-catenin in prostate cancer cells. Scale bar, 10 μm. **h** CHIP assay suggested that β-catenin could bind to the promoter region of *STAT3*, while the specific inhibitor of β-catenin (XAV-939) partly decreased their binding activity. **i** CCR5 specific siRNAs could significantly abrogate the activation effect of CCL5 on β-catenin/STAT3 pathway (*n* = 3). All values are presented as the mean ± SD, *p < 0.05, **p < 0.01.

Uncovering the underlying mechanism for CCL5-induced STAT3 activation might provide potential therapeutic targets for prostate cancer. RNA-Seq analysis was conducted to characterize the cellular responses of PC3 cells to CCL5 treatment. Differential expression gene analysis showed that 94 metastasis-related genes, 42 stemness-related genes as well as 30 STAT3 pathway-related genes were upregulated higher than 2 folds (log_2_^FC^ > 1, *P* < 0.05) changes in PC3 cells after CCL5 treatment. By intersecting the three data sets, we identified a cluster containing 4 genes in common, while CTNNB1, also known as β-catenin, was the most significant one of them (Fig. 5a–d). Western blotting assay indicated that the CD133^+^ PCSCs sorted by MACS method exhibited increased expression of CCR5 (the binding receptor of CCL5), β-catenin and STAT3 when compared with the CD133^-^ subpopulation, suggesting that CCL5 might promote prostate cancer metastasis by binding with CCR5 in PCSCs and then promote PCSCs self-renewal via activating the β-catenin and STAT3 pathway (Fig. 5e). In order to clearly uncover the mechanisms of CCL5 in promoting PCSCs, we further investigated the interaction between β-catenin and STAT3 in the presence of CCL5 treatment. As shown in Fig. 5f, CCL5 treatment significantly increased the promoter activity of *STAT3* in PC3 cells while XAV-939, the specific inhibitor of β-catenin, partly abrogated that. Furthermore, CCL5 treatment also significantly induced the expression and nuclear translocation of β-catenin in prostate cancer cells (Fig. 5g). These results indicated that CCL5 might activate STAT3 transcription by elevating β-catenin expression and its binding to the promoter region of *STAT3*. To convince this speculation, the direct interaction between β-catenin protein and *STAT3* gene was investigated. The −574 to −560 promoter region of STAT3 was predicted as the binding site of β-catenin using JASPAR database. Additionally, CHIP assay suggested that β-catenin could bind to the predicted promoter region of *STAT3*, while the specific inhibitor of β-catenin (XAV-939) partly decreased their binding activity (Fig. 5h). Finally, Western blotting assay convinced the crucial role of CCR5 in CCL5-induced β-catenin/STAT3 pathway activation as CCR5 specific siRNAs could significantly abrogate the activation effects of CCL5 on β-catenin/STAT3 pathway (Fig. 5i). Altogether, these results indicated that CCL5 could promote prostate cancer invasion and PCSCs self-renewal *via* activating the CCR5/β-catenin/STAT3 pathway.

### CCL5 knockdown in TAMs suppresses prostate cancer growth, bone metastasis and PCSCs activity in vivo

**Fig. 6:**
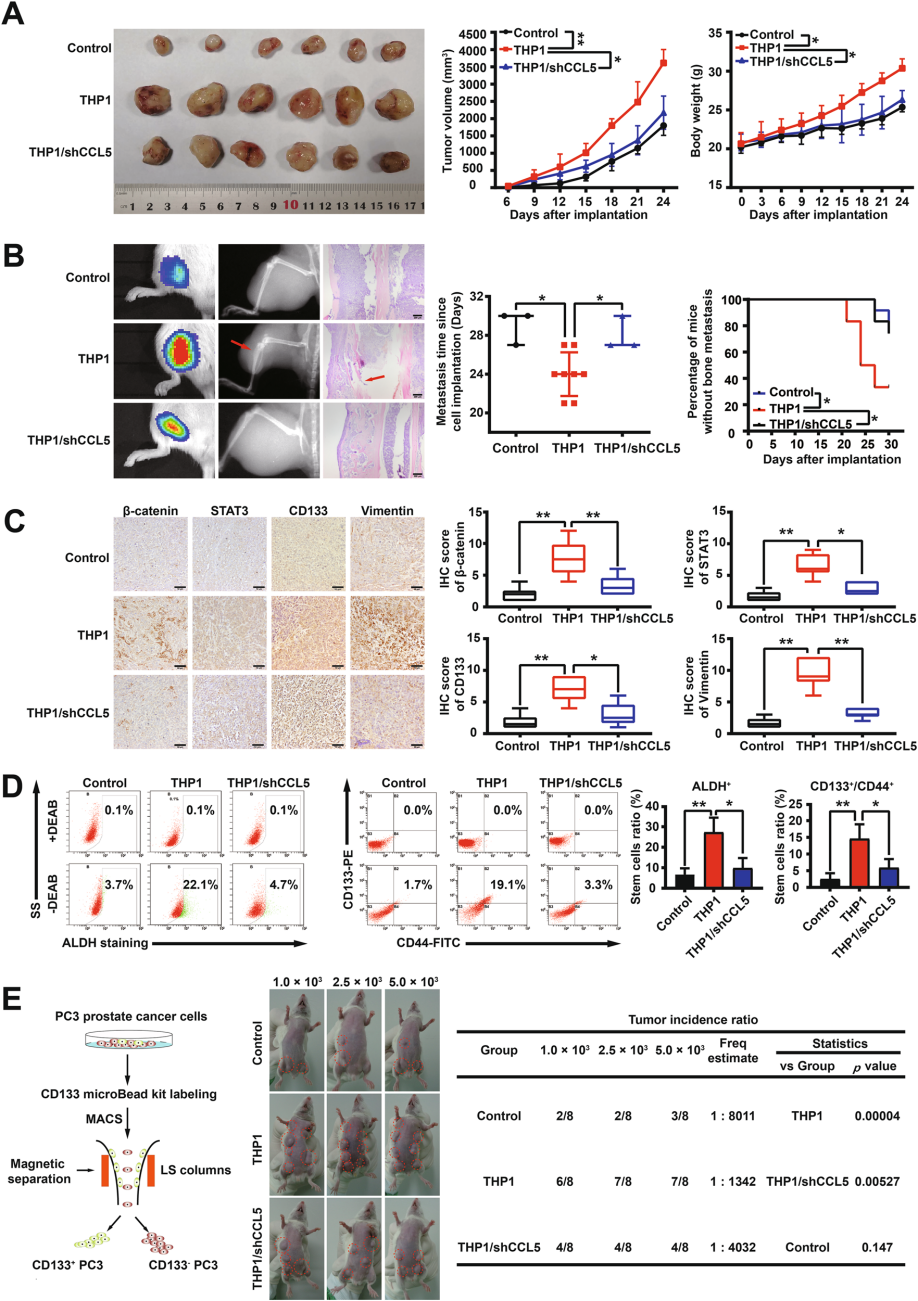
CCL5 knockdown in TAMs suppresses prostate cancer growth, bone metastasis and PCSCs activity in vivo. **a**, **b** THP1-derived TAMs co-injection not only significantly accelerated PC-3-Luc xenografts growth but also increased the bone metastasis of prostate cancer xenografts, whereas CCL5 knockdown in THP1-derived TAMs could partly abrogate that. Mice were imaged using the IVIS Lumina XR in vivo imaging system to monitor PC3-Luc xenografts growth and bone metastasis (*n* = 12). Scale bar, 200 μm. **c** Immunohistochemistry indicated that CCL5 knockdown in THP1-derived TAMs could also significantly abrogate the up-regulation effect of TAMs on metastasis and stemness-related markers including β-catenin, STAT3, CD133, and Vimentin in PC-3-Luc xenografts (*n* = 6). Scale bar, 50 μm. **d** CCL5 knockdown in THP1-derived TAMs could also significantly abrogate the promotion effect of THP1-derived TAMs on PCSCs self-renewal activity in vivo. Primary cells were isolated from fresh tumors by mechanical methods and subjected to PCSCs subpopulation analysis (*n* = 3). **e** In vivo limiting dilution assay was conducted to compare the tumorigenicity changes of PCSCs after CCL5 knockdown in co-cultured TAMs. The tumorigenic cell frequency estimate and *p* values were calculated by the limiting dilution analysis tool (http://bioinf.wehi.edu.au/software/elda/) (*n* = 8). *p < 0.05, **p < 0.01.

To investigate the effect of TAMs-secreted CCL5 on prostate cancer metastasis and PCSCs activity in vivo, PC-3-Luc cells and THP1-derived TAMs with different CCL5 expression levels were co-injected subcutaneously into the tibia regions of NOD/SCID mouse. As shown in Fig. 6a, b, THP1-derived TAMs co-injection not only significantly accelerated PC-3-Luc xenografts growth but also increased the bone metastasis of prostate cancer xenografts, whereas CCL5 knockdown in THP1-derived TAMs significantly abrogated that. Furthermore, no mouse deaths or other adverse effects were observed during the animal experiment. The decreased mice weights in TAMs/shCCL5 group compared with the TAMs group may be attributed to the decreased tumor burden. Moreover, consistent with the in vitro results, CCL5 knockdown in THP1-derived TAMs could also significantly abrogate the upregulation effect of TAMs on metastasis and stemness-related markers including β-catenin, STAT3, CD133, and Vimentin (Fig. 6c). Notably, CCL5 knockdown in THP1-derived TAMs significantly abrogated the promotion effects of THP1-derived TAMs on PCSCs self-renewal activity in vivo (Fig. 6d). Tumorigenesis assay is the “golden standard” for identifying CSCs activity^30^. To further convince the promotion effect of TAMs-secreted CCL5 on PCSCs activity, in vivo limiting dilution assay was conducted to compare the tumorigenicity changes of PCSCs after CCL5 knockdown in co-cultured TAMs. As shown in Fig. 6e, PCSCs sorted from the PC3 and TAMs co-culture group initiated tumors with a higher incidence in NOD/SCID mice when compared with the control PCSCs, whereas CCL5 knockdown in TAMs could partly decrease that. Altogether, CCL5 knockdown in TAMs could suppress prostate cancer xenografts growth, bone metastasis as well as PCSCs self-renewal and tumorigenicity in vivo.

### CCL5 expression is positively correlated with advanced clinicopathological characteristics and poor prognosis of prostate cancer

**Fig. 7:**
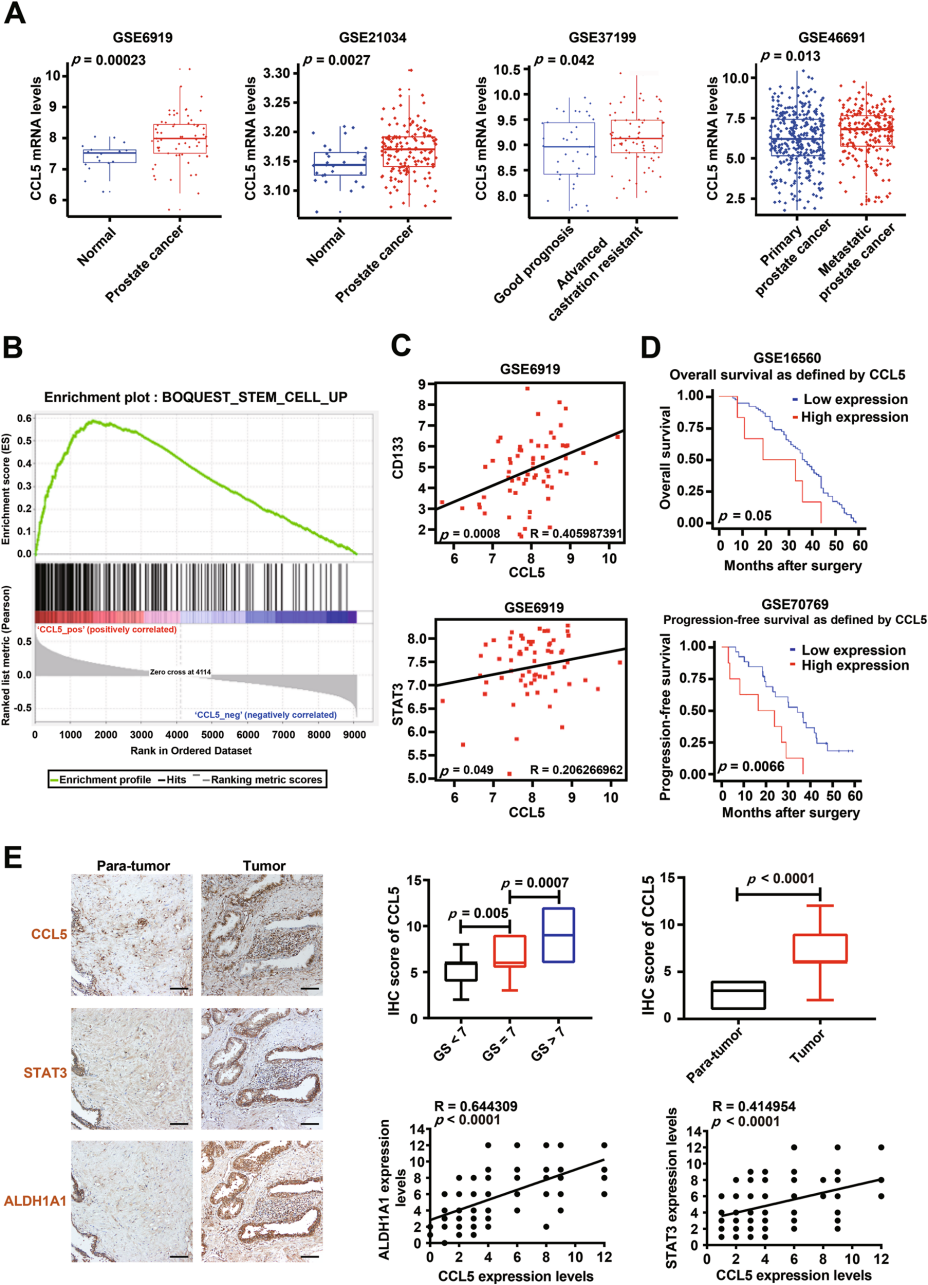
The clinical significance of CCL5 in predicting prostate cancer prognosis. **a** The CCL5 mRNA expression differences between different tissues or different groups of prostate cancer patients. CCL5 expression increased in prostate cancer tumor tissues when compared with nonmalignant prostate tissues. Castration-resistant prostate cancer patients exhibited increased CCL5 expression when compared with patients with good prognosis. Metastatic prostate tumors exhibited increased CCL5 expression when compared with primary prostate tumors. The following datasets were analyzed including GSE6919 (*n* = 93), GSE21034 (*n* = 179), GSE37199 (*n* = 106), and GSE46691 (*n* = 545). **b** Enrichment analysis indicated that CCL5 was positively correlated with stem cell-related genes. **c** There were significant positive correlations between the expression levels of CCL5 and stemness-related genes including CD133 and STAT3 (*n* = 93). **d** High CCL5 expression in prostate cancer patients predicted poor overall survival (*n* = 82) and poor progression-free survival (*n* = 34). **e** A commercialized prostate tissue microarray containing 90 prostate cancer tissues and 90 para-carcinoma tissues was analyzed. Prostate cancer patients with high Gleason grade exhibited increased CCL5 expression (*p* < 0.05). CCL5 overexpression was observed in prostate cancer tissues when compared with para-carcinoma tissues (*p* < 0.05). There were significant positive correlations between CCL5 and PCSCs-related proteins including ALDH1A1 and STAT3 (*n* = 180) in the samples of human prostate cancer tissue microarray. Scale bar, 100 μm.

Finally, the clinical significance of CCL5 for prostate cancer patients was investigated. Consistent with our finding, bioinformatic analysis results indicated that CCL5 mRNA expression was increased in prostate cancer tumor tissues when compared with nonmalignant prostate tissues (*p* < 0.01). Furthermore, castration-resistant prostate cancer patients also exhibited increased CCL5 expression when compared with patients with good prognosis (*p* = 0.042). Moreover, metastatic prostate tumors also exhibited increased CCL5 expression when compared with primary prostate tumors (*p* = 0.013) (Fig. 7a). Enrichment analysis indicated that CCL5 was positively correlated with stem cell-related genes (Fig. 7b). Additionally, there were significant positive correlations between the expression levels of CCL5 and stemness-related genes including CD133 and STAT3 (*p* < 0.05) (Fig. 7c). More importantly, high CCL5 expression in prostate cancer patients usually predicated poor overall survival (*p* = 0.05) and poor progression-free survival (*p* = 0.0066) (Fig. 7d). To further convince the clinical significance of CCL5 for prostate cancer patients, a commercialized prostate tissue microarray containing 90 prostate cancer tumor tissues and 90 para-carcinoma tissues were analyzed (Fig. 7e). It was found that prostate cancer patients with high Gleason grade (GS > 7) exhibited increased CCL5 expression (*p* < 0.05). Additionally, CCL5 overexpression was observed in prostate cancer tissues when compared with para-carcinoma tissues (*p* < 0.05). Notably, the significant positive correlations between CCL5 and PCSCs-related proteins including ALDH1A1 (*p* = 0.0001) and STAT3 (*p* = 0.0001) were observed in the samples of human prostate cancer tissue microarray. Altogether, high CCL5 expression may predict poor prognosis and increased PCSCs activities in prostate cancer patients.

## Discussion

Prostate cancer is the most frequently diagnosed cancer among males in 105 countries and the leading cause of cancer death among men in 46 countries worldwide^1^, mainly in European countries and North America. Many patients with no evidence of metastatic disease undergo treatment for cure with surgery or radiation. For advanced and metastatic prostate cancer, the therapeutic focus has largely revolved around androgen deprivation therapy (ADT) which usually achieves short-term effectiveness but ultimately induces drug resistance and metastasis^10,31^. Increasing evidence has indicated that ADT induces the enrichment of PCSCs subpopulation, which are resistant to traditional killing strategies and drive subsequent metastasis^32–34^. Therefore, eliminating PCSCs is crucial for improving the clinical outcomes of prostate cancer. Although many studies have examined the mechanisms underlying the expansion and self-renewal of PCSCs, direct targeting strategies against PCSCs are disappointing in vivo, which might be owing to the complex signaling and the heterogeneous TME network^10^.

CSCs require a niche that provides a specific microenvironment supporting their self-renewal^35^. As the key component in TME, TAMs have attracted increased attention in the field of CSCs regulation owing to their phenotypic and functional plasticity. Recent studies have indicated that CSCs prefer to remodel their specific niche by recruiting monocytes and educating them to become TAMs^36^. Subsequently, the infiltrated TAMs within the CSCs niche produce cytokines that reciprocally facilitate the expansion and self-renewal of CSCs^37^. For example, it was reported that breast CSCs could recruit TAMs into the CSCs niche through secreting hyaluronan synthase 2 (HAS2) and then lead to enhanced secretion of platelet-derived growth factor-BB from TAMs, which finally enhanced the self-renewal of breast CSCs^37^. Additionally, numerous reports have suggested that TAMs were necessary for facilitating CSCs evading from the continuous immune surveillance in multiple malignancies^38–40^. In terms of prostate cancer, the interaction between TAMs and PCSCs as well as the in-depth mechanisms remain largely unknown. In the present study, for the first time, we proved that TAMs/CCL5 could promote PCSCs self-renewal and metastasis via activating β-catenin/STAT3 signaling. This finding was consistent with the existing knowledge that TAMs could facilitate the progression and therapy resistance of prostate cancer by promoting the self-renewal of PCSCs^10^. More significantly, CCL5 knockdown in TAMs by genetic approaches suppressed prostate cancer growth, bone metastasis and PCSCs activity in vivo. These findings establish a strong rationale for developing TAMs/CCL5 as a novel therapeutic target to inhibit PCSCs and prostate cancer metastasis. Penn et al. also used to report that specific elimination of TAMs in murine models of ovarian cancer by TAMs-targeted nanoparticles could significantly decrease the CSCs population and therefore inhibit ovarian cancer growth^41^. Altogether, targeting TAMs may be a promising and powerful strategy to eliminate CSCs which deserves further investigations.

As CSCs self-renewal closely depends on the supportive effect of TAMs, elucidating the molecular steps by which TAMs promote PCSCs self-renewal may provide potential therapeutic targets for PCSCs elimination. It is generally accepted that TAMs could secrete a series of soluble cytokines as inflammatory mediators that influence cancer progression by recruiting and activating various immunosuppressive cells to the tumor stroma and finally promoting carcinogenesis and metastasis^20^. For example, TAMs-secreted CXCL1 could recruit myeloid-derived suppressor cells (MDSCs) into the TME, which secreted chemokines including S100A8/9 that enhanced cancer cell survival, chemoresistance, metastasis, and pre-metastatic niche formation^42,43^. In fact, TAMs-derived cytokines could also directly act on the surface receptors in CSCs membrane through autocrine or paracrine, and promote the survival and self-renewal of CSCs^14^. For example, Yang et al. reported that TAMs could promote the self-renewal of murine breast CSCs by releasing EGF and therefore activate the paracrine EGFR/STAT3/Sox2 signaling in breast CSCs^44^. Wan et al. reported that TAMs-derived IL-6 could increase the CD44^+^ liver CSCs subpopulation and enhance their sphere formation capacities by activating the STAT3 signaling^45^. To our best knowledge, the molecular mechanism by which TAMs modulate PCSCs has been rarely explored. In the present study, by conducting RNA-seq and CHIP assays, we demonstrated that TAMs could promote PCSCs self-renewal by releasing stemness-favoring cytokine CCL5 which bound with its receptor CCR5 in PCSCs and thus promoting β-catenin expression as well as its binding to the promoter of *STAT3* in PCSCs. These results are consistent with the existing report that TAMs could significantly promote prostate cancer progression and ADT resistance via activating the STAT3 pathway^10^. Given CCL5 is highly expressed by TAMs rather than prostate cancer cells, the combinational therapeutic strategy of blocking TAMs and neutralizing CCL5 may be a promising and powerful approach for PCSCs elimination in the future.

Chemokines are a large family of small cytokines with molecular weights ranging from 7 to 15 kDa. To date, approximately 50 chemokines have been identified^46^ and several TAMs-derived chemokines have been reported to be associated with cancer progression and metastasis. For example, it has been reported that TAMs could promote prostate cancer migration through activation of the CCL22-CCR4 axis^32^. TAMs released CCL18 to further promote the aggressive phenotype of breast phyllodes tumors by enhancing and maintaining the myofibroblast differentiation and invasion in vitro and in vivo^23^. Our previous study indicated that CCL5 was one of the most abundant chemokines secreted by TAMs^22^. It has been reported that CCL5 secreted by TAMs may promote the proliferation, invasion and metastasis of gastric cancer cells, in which STAT3 signaling is likely to play an important role^47^. However, the role and prognosis significance of CCL5 in prostate cancer have been rarely reported so far, and little is known about the influence of TAMs-secreted CCL5 on PCSCs. In the present study, we demonstrated that high CCL5 expression was significantly correlated with high Gleason grade, poor prognosis, metastasis as well as increased PCSCs activity in prostate cancer patients by conducting clinical investigations and bioinformatic analysis. Further studies identified TAMs rather than prostate cancer cells as the major source of CCL5. More importantly, CCL5 knockdown in TAMs could dramatically suppress PCSCs self-renewal and prostate cancer growth and metastasis. It has been reported that the CCL5-CCR5 axis blockage by maraviroc, an FDA-proved CCR5 inhibitor, could dramatically suppress tumor growth in a murine PDX model of human breast phyllodes tumor^23^. Therefore, our finding not only identified CCL5 as a potential biomarker for prostate cancer prognosis and metastasis but also suggested that targeting TAMs/CCL5 may represent a potential clinically available strategy for inhibiting PSCSs and prostate cancer metastasis. As PCSCs express high levels of CCR5 (CCL5 receptor) than non-stem prostate cancer cells, developing PSCSs-targeting drug delivery system loaded with CCR5-specific small molecular inhibitors (i.g. maraviroc) to block the CCL5-CCR5 axis in PCSCs may represent a potential treatment strategy to eliminate PCSCs. Additionally, TAMs were identified as the major cell type of CCL5 secretion in the present study. In consideration of the emerging TAMs-targeting nanomaterials in recent years^48^, the TAMs-targeting nanomaterials loaded with CCL5 neutralizing antibody may be a more selective and powerful strategy to suppress the TAMs/CCL5 activity.

In summary, the present study demonstrates that TAMs-secreted CCL5 could promote PCSCs self-renewal and prostate cancer metastasis via activating β-catenin/STAT3 signaling. This study not only uncovers the modulatory mechanism of TAMs/CCL5 in promoting PCSCs self-renewal and prostate cancer metastasis but also provides a novel rationale for developing TAMs/CCL5 as a potential molecular target for PCSCs elimination and metastatic prostate cancer prevention.

## Materials and methods

### Cell culture and induction

Human monocyte cell line THP1 and human prostate cancer cell lines DU145, PC3, LnCaP, and VCaP were purchased from the American Type Culture Collection (ATCC, Maryland, USA). All the above cell lines were authenticated by short tandem repeat profiling and tested for mycoplasma contamination. The PC3-Luc cells, fluoresced in the presence of d-luciferin substrate, were obtained by transfecting PC3 cells with the lentiviral luciferase reporter plasmid. All the cells were cultured in RPMI-1640 or DMEM mediums supplemented with 10% fetal bovine serum, 1% penicillin and streptomycin (Gibco, Grand Island, NY, USA), and maintained at 37 °C in a humidified incubator containing 5% CO_2_. THP1 monocytes were treated with 100 ng/ml phorbol-12-myristate-13-acetate (PMA, Sigma-Aldrich, Shanghai, China) for 24 h to induce their attachment and differentiation into macrophages. Subsequently, THP1-derived macrophages were transformed into M2 phenotype (TAMs) after 10 ng/ml IL-4 induction (Sigma-Aldrich) for 72 h^22^. The successful induction was verified by detecting the M2 phenotype macrophage markers including CD163 and Arg1 using Flow cytometry or Western blotting. The cell culture supernatant of THP1-derived TAMs was collected as conditioned medium (CM) and was used in further studies as indicated.

### Cell viability assay

The effects of CCL5 on the proliferation of prostate cancer cells were investigated using CCK-8 method. In brief, DU145 and PC3 cells were seeded in 96-well plates at a density of 3 × 10^3^ cells per well. After attachment, cells were treated with serial concentration gradients of recombinant human CCL5 (PeproTech, Rocky Hill, USA) for 24, 48, or 72 h. Then, cell viability was detected using CCK-8 kit (KeyGEN BioTECH, Nanjing, China) according to the manufacturer’s instructions.

### Colony formation assay

Effects of CCL5 on the colony formation abilities of prostate cancer cells were investigated by colony formation assay. Prostate cancer cells were seeded in 6-well plates at a density of 500 cells per well. After attachment, cells were treated with 20 or 40 ng/ml CCL5. The resultant colonies were fixed with 4% paraformaldehyde and then stained with 0.1% coomassie blue solution.

### Wound healing and transwell assay

Wound healing assay and transwell assay were conducted to measure the migration and invasion ability changes of prostate cancer cells after exogenous interventions as indicated, respectively. For wound healing assay, prostate cancer cells grown in 6-well plates as confluent monolayers were mechanically scratched using a 1 ml pipette tip to create the wound. Subsequently, cells were washed with PBS solution twice, refreshed with complete medium or TAMs CM, and treated with recombinant human CCL5 or CCL5 neutralizing antibody (NA) as indicated. Wound gaps were microscopically recorded and the wound gap distances were compared among groups. For transwell assay, 100 μl matrigel (354248, Corning, NY, USA) was smoothly spread on the upper chamber (8 μm pore size, Milipore, Billerica, MA, USA) of the 24-well transwell insert to simulate the basement membrane, and then incubated overnight at 37 °C for gelling. Subsequently, prostate cancer cells were serum-starved for 24 h and then seeded in the matrigel-coated chambers at a density of 5 × 10^4^ cells/chamber with serum-free medium or TAMs CM. Recombinant CCL5 or CCL5 NA were added to the upper chambers as indicated while the lower chamber of the transwell inserts was filled with 600 μl complete medium. After 24 h treatment, the non-invaded cells on the top of the upper chamber were scraped off with a cotton swab while the invaded cells on the bottom of the upper chamber were stained with 0.1% coomassie blue solution.

### QPCR

Total RNA was extracted using RNAiso Plus (9109, TaKaRa, Tokyo, Japan). After removing genomic DNA by gDNA eraser, total RNA was reversely transcribed to complementary cDNA using the PrimeScript™RT reagent Kit (RR047A, TaKaRa). cDNA amplification was performed using the SYBR® Premix Ex Taq™ II KIT (RR820A, TaKaRa) and the ABI Quant Studio 7 Flex Real-Time PCR System (Applied Biosystems, Foster City, USA). The assay procedure for each kit was conducted according to the manufacturer’s instructions. The 2^−ΔΔCt^ method was used to calculate the relative expression levels of genes among groups. The primer sequences were presented in Supplementary Table 1.

### Western blotting

Western blotting assay was conducted as we have previously reported^49^. The primary antibodies included MMP2 (A6247), STAT3 (A11216) (Abclonal, Wuhan, China), p-STAT3 (9145), GAPDH (5174), iNOS (39898), MMP9 (13667) (Cell Signaling Technology, CST, Boston, MA, USA), β-catenin (17565-1-AP), β-actin (20536-1-AP), E-cadherin (20874-1-AP), N-cadherin (22018-1-AP), Vimentin (10366-1-AP) (Proteintech Group, Chicago, IL, USA), CCR5 (AF6339), CCL5 (AF5151), Arg1 (DF6657) (Affinity Biosciences, Cincinnati, OH, USA).

### Plasmid transfection and siRNA interference

The lentiviral luciferase plasmid was purchased from GeneCopoeia (LPP-Hluc-Lv201-100, GeneCopoeia, Guangzhou, China) and transfected into PC3 cells according to the manufacturer’s protocol. The commercialized recombinant plasmid and shRNA plasmid for CCL5 as well as the non-targeting control plasmid were purchased from Vigene Biosciences (Jinan, China). The commercialized siRNAs targeting CCR5 as well as the scramble siRNAs were purchased from Transheep Biosciences (Shanghai, China). All the above plasmids or siRNAs were transfected into the indicated cells using Vigenefection (FH880806, Vigene Biosciences) according to the manufacturer’s instructions.

### Mammosphere formation assay

For mammosphere formation assay, prostate cancer cells were seeded in 6-well ultralow attachment plates at a density of 2 × 10^4^ cells per well. After cell attachment, the medium was refreshed with DMEM/F12 medium supplemented with 1% penicillin-streptomycin (Gibco), 2% B27 (Gibco), 0.4% bovine serum albumin, 20 ng/ml EGF and 5 μg/ml insulin (Sigma). Prostate cancer cells were treated as indicated. The number and size of mammospheres were quantified microscopically.

### Macrophage polarization analysis

Macrophages were incubated with FITC-conjugated F4/80 antibody (SC-71085, Santa Cruz, Dallas, TX, USA) and PE-conjugated CD163 antibody (12-1639-42, eBioscience, San Diego, CA, USA) for 30 min at 37 °C. The F4-80^+^/CD163^+^ subpopulation was quantified by flow cytometry using the FC500 flow cytometer (Beckman Coulter, California, USA) and defined as M2 phenotype macrophages (TAMs).

### PCSCs population analysis and sorting

For PCSCs population analysis, prostate cancer cells were incubated with FITC-conjugated CD44 antibody (11-0441-82, BD Biosciences, San Diego, CA, USA) and PE-conjugated CD133 antibody (372804, Biolegend, San Diego, CA, USA) for 30 min at 37 °C. The CD44^+^/CD133^+^ subpopulation was quantified by flow cytometry and defined as PCSCs. Hyperactive aldehyde dehydrogenase (ALDH) activity is closely related to the physiological properties of CSCs^50^. PCSCs population analysis was also conducted by flow cytometry using the ALDEFLUOR Stem Cell Identification Kit (01700, STEMCELL Technologies, Vancouver, Canada) according to the manufacturer’s instructions. The ALDH^+^ subpopulation was quantified and defined as PCSCs. As a specific inhibitor of ALDH activity, diethylaminobenzaldehyde (DEAB), was used to control for background fluorescence in the ALDH staining assay. For PCSCs sorting, the CD133^+^ subpopulation was isolated by the magnetic activated cell sorting (MACS) method using the CD133 MicroBead Kit (130-100-857, Miltenyi, North Rhine-Westphalia, Germany) in accordance with the manufacturer’s instructions.

### Patients and specimens

The blood samples, prostate cancer tumor tissues, the corresponding paracancerous tissues and lymph node metastatic lesions as well as their clinicopathological data were collected in Guangdong Provincial Hospital of Chinese Medicine in 2019. For the control group, blood specimens were collected from 30 healthy males selected at random. The institutional review board approval was obtained from the Ethics Committee of Guangdong Provincial Hospital of Chinese Medicine (NO. BF2019-050-01). Prior to sample collection, all participants were informed of the purpose of the study and had provided the written informed consent. The expression difference of CCL5 between tumor tissues and paracancerous tissues of prostate cancer patients was investigated by both QPCR and Western blotting methods. Serums were separated from blood samples and serum CCL5 concentrations were determined using a commercially available CCL5 ELISA kit (SEA116Hu, Cloud-Clone Corp, Wuhan, China) according to the manufacturer’s instructions. Serum CCL5 concentration differences between healthy males and prostate cancer patients were compared. Additionally, Gleason scores (GS) of prostate cancer patients were evaluated according to the eighth edition of Gleason Score Classification Guideline from the American Joint Committee on Cancer (AJCC). In the present study, 30 prostate cancer patients were pathologically classified into three groups including GS < 7 (*n* = 5), GS > 7 (*n* = 14), and GS = 7 (*n* = 11). Serum CCL5 concentration differences in prostate cancer patients with different GS levels were also compared. Tissue immunofluorescence assay was conducted to investigate the colocalization of CCL5 and the macrophage marker CD163 in the tissue specimens of prostate cancer patients.

### Elisa assay

CCL5 concentrations in the cell culture supernatants of THP1-derived macrophages (M0), THP1-derived TAMs (M2), DU145, and PC3 cells were measured using a commercially available CCL5 ELISA kit (SEA116Hu, Cloud-Clone Corp) according to the manufacturer’s instructions.

### Immunofluorescence assay

Cell immunofluorescence assay was conducted to investigate the effect of CCL5 on STAT3 and β-catenin transportation. DU145 and PC3 cells were firstly seeded in the confocal dish at a density of 3 × 10^4^ cells per well. After 40 ng/ml CCL5 treatment for 24 h, cells were fixed with 4% paraformaldehyde for 20 min, washed three times with PBS and then permeabilized with 0.25% Triton X-100 for 20 min. After blocking with 5% BSA for 1 h, cells were incubated with STAT3 antibody (A11216, Abclonal) or β-catenin antibody (8480S, CST) overnight at 4 °C. Subsequently, cells were incubated with Alexa Fluor® 555 conjugated Anti-rabbit IgG (4413S, CST) at room temperature for 1 h in the dark. Subsequently, the nucleus was visualized by 0.1% 4′,6-diamidino-2-phenylindole (DAPI, Sigma-Aldrich) at room temperature for 15 min. Fluorescence images were captured using the LSM710 confocal microscope (Zeiss, Jena, Germany). Additionally, tissue immunofluorescence assay was conducted as we previously described^22^ to investigate the co-localization of CCL5 and the macrophage marker CD163 in the primary tumors and metastatic lymph nodes of prostate cancer patients. The primary antibodies including CCL5 antibody (DF7427, Affinity) and PE-conjugated CD163 antibody (12-1639-42, eBioscience) as well as the secondary antibody of Alexa Fluor® 488 conjugated Anti-rabbit IgG (4412S, CST) were used in the tissue immunofluorescence assay.

### Immunohistochemistry (IHC) and hematoxylin-eosin staining (HE)

IHC assay was conducted using the Polymer Detection System For lmmunohistological Staining (PV-9000, Zhongshanjinqiao Biotech) according to the manufacturer’s instructions. The primary antibodies against β-catenin (17565-1-AP, Proteintech), Vimentin (10366-1-AP, Proteintech), STAT3 (60199-1-Ig, Proteintech), CD133 (18470-1-AP, Proteintech), CCL5 (AF5151, Affinity), ALDH1A1 (BF0220, Affinity) and peroxidase-conjugated goat antirat IgG (ZB-2307, Zhongshanjinqiao Biotech) were used in the IHC experiment. Hematoxylin and Eosin staining was conducted using the Hematoxylin and Eosin Staining Kit (C0105, Beyotime Biotechnology, Shanghai, China) according to the manufacturer’s instructions. In short, tissue slides were deparaffinized firstly. Then, 10% hematoxylin was used to visualize the cell nucleus, while 1% eosin was used to stain the cytoplasm. Digital images of stained sections were captured using the BX53 upright metallurgical microscope (Olympus, Center Valley, PA, USA).

### Tissue microarray analysis

A commercialized human prostate cancer tissue microarray (HProA180PG05, Outdo Biotech, Shanghai, China) was used to analyze the correlation between CCL5 expression and PCSCs-related proteins. Briefly, the expression levels of CCL5, STAT3, and ALDH1A1 in prostate cancer tissue specimens were determined by the IHC method using primary antibodies against CCL5 (AF5151, Affinity), STAT3 (60199-1-lg, Proteintech) and ALDH1A1 (BF0220, Affinity). Subsequently, the expression levels of the above proteins were quantified by two experienced pathologists using the immune response score (IRS) method^10^. Both the staining area and the staining intensity were scored semi-quantitatively, which were multiplied to obtain the final IRS scores. Spearman’s rank correlation coefficient was used to analyze the correlation between CCL5 expression and PCSCs-related protein expression.

### RNA sequencing

To define the gene expression changes of PC3 cells in response to 40 ng/ml CCL5 treatment for 48 h, high-throughput RNA sequencing (RNA-Seq) was conducted by Genedenovo Biotechnology Co., Ltd (Guangzhou, China) on the HiSeqTM 4000 sequencing platform (Illumina, California, USA). Enrichment of the mRNA, fragment interruption, the addition of adapters, size selection, PCR amplification and RNA-Seq were performed as we have previously reported^49^. The gene abundances were calculated and normalized by the RPKM (Reads Per kb per Million reads) method^51^. The edgeR package (http://www.r-project.org/) was used to identify differentially expressed genes (DEGs) between control and CCL5-treated groups. Genes with a fold change (FC) ≥ 1 and *p* value < 0.05 in comparison were identified as significant DEGs.

### Chromatin immunoprecipitation (ChIP) assay

To investigate whether CCL5 treatment could promote the binding of β-catenin to the promoter region of *STAT3* in prostate cancer cells, ChIP assay was performed by immune-precipitating the DNA fragments with β-catenin antibody (8480S, CST) using the ChIP Assay Kit (P2078, Beyotime, Shanghai, China) according to the manufacturer’s instructions. The anti-human IgG was used as negative control. The −574 to −560 promoter region of *STAT3* was predicted as the binding site of β-catenin using JASPAR database (http://jaspar.genereg.net/). This region in the immune-precipitated DNA samples was amplified by PCR method using the following primers: 5′-AAGTGATGGAACGGAGTACGG-3′ (forward) and 5′-TCTTACCACGCGGGAATCAG-3′ (reverse).

### Double luciferase reporter gene assay

STAT3 promoter activity changes of prostate cancer cells when treated with different stimuli were investigated by double luciferase reporter gene assay. Briefly, the CCL5 promoter plasmid (HPRM44745-PG04, GeneCopoeia) was transfected into prostate cancer cells using Vigenefection (FH880806, Vigene Biosciences) according to the manufacturer’s instructions. Then, the transfected cells were seeded in the 96-well plate at a density of 1 × 10^4^ cells/well and treated as indicated. Subsequently, the cell culture supernatant was collected. The STAT3 promoter activity was detected by analyzing the gaussia luciferase activity and secreted alkaline phosphatase activity in the cell culture supernatant using the Secrete-Pair™ Dual Luminescence Assay Kit (LF031, Genecopeia) according to the manufacturer’s instructions.

### Bioinformatic analysis

Gene set enrichment analysis (GSEA) was applied to analyze the function and pathway of CCL5. Genome-wide expression profiles from tumor samples were used to rank all genes in the data set, and the ranking list was then used to calculate enrichment score (ES) and *p* value. The procedure included obtaining the gene-ranking list, calculating an ES, estimating the significance level of the ES, and correcting the significance level for multiple gene sets. Details of the steps of the GSEA procedure has been implemented in JAVA and R, and different versions of GSEA packages can be downloaded at www.broadinstitute.org/gsea/index.jsp. All data analyses were performed in the R programming environment (version 3.5.2) and Bioconductor. Survival analysis was performed according to the Kaplan-Meier analysis and log-rank test. Overall survival (OS) was defined as the time between the date of surgery and date of death or the date of the last follow-up.

### Animal experiments

The in vivo experiments were performed according to our institutions’ guidelines for the use of experimental animals and were approved by the Institutional Animal Care and Use Committee of Guangdong Provincial Hospital of Chinese Medicine (No. 2018071). The 4-week old male NOD/SCID mice were raised in the Experimental Animal Center of Guangdong Provincial Hospital of Chinese Medicine under specific pathogen-free conditions and given sterilized food and water. For PC3-Luc xenograft assay, mice were randomized into three groups using the random number table method. Then, 3 × 10^6^ PC3-Luc cells were injected subcutaneously into the tibia regions of mice in the control group, while a co-injection of 3 × 10^6^ PC3-Luc cells with 9 × 10^6^ THP1-derived TAMs or THP1/shCCL5-derived TAMs was performed in mice of the experimental group (*n* = 6 in each group). Throughout the treatment, mice were weighed and their tumors were measured with a caliper every 3 days. Tumor volumes (V) were calculated using the formula: *V* = (length × width^2^)/2. d-Luciferin (150 mg/kg weight, 122799, PerkinElmer, Boston, USA) was injected intraperitoneally and mice were imaged using the IVIS Lumina XR in vivo imaging system (PerkinElmer) every week to monitor PC3-Luc xenografts growth and bone metastasis. When tumors grew to a proper size or mice survived to indicated days, mice were euthanized and tumors were excised. Primary cells were isolated from fresh tumors by mechanical methods and subjected to PCSCs subpopulation analysis as indicated above. The remaining tumor tissues were stored at −80 °C and used for IHC assay as indicated above. The tibias of mice were also excised and subjected for HE assay as indicated above. For PCSCs tumorigenicity assay, the CD133^+^ PCSCs were firstly sorted from PC3 cells by MACS method as indicated above. Subcutaneously, PCSCs were diluted in the appropriate cell doses as indicated, and then resuspended in matrigel alone or resuspended with THP1-derived TAMs or THP1/shCCL5-derived TAMs at a ratio of 1:3. Subcutaneously, the resuspended cells were injected subcutaneously into the abdomen and chest areas of NOD/SCID mice. The number of tumors formed from each cell dose injected was scored. The tumorigenic cell frequency estimate and *p* values were calculated by the limiting dilution analysis tool (http://bioinf.wehi.edu.au/software/elda/)^10^. No blinding method was needed in the animal experiments.

### Statistical analyses

Statistical analyses were performed using the SPSS 22.0 software (Abbott Laboratories, Chicago, USA). To ensure adequate power to detect a pre-specified effect, the sample size was chosen using the Power and Sample Size Program (http://biostat.mc.vanderbilt.edu/PowerSampleSize). Data were represented as the mean ± standard deviation and analyzed pairwise for statistical significance using one-way analysis of variance or Student’s *t*-test. Levene’s test of equality of variances was used to assess the assumption of homogeneity of variance. Spearman’s rank correlation coefficient was used to analyze the correlation between CCL5 expression and stemness-related proteins. Survival curve was plotted by the Kaplan-Meier method and compared using the log-rank analysis. All tests were two-sided and *p*-values less than 0.05 were considered statistically significant.

## Supplementary information

Supplementary Table 1

Supplementary Figure 1

Supplementary Figure Legends
