# Supplementary material for: CCL5 derived from tumor-associated macrophages promotes prostate cancer stem cells and metastasis via activating β-catenin/STAT3 signaling
Source: Cell Death Dis. 2020 Apr 16;11(4):234. doi: 10.1038/s41419-020-2435-y (PMC7162982; doi:10.1038/s41419-020-2435-y)
Supplement: Supplementary file 1 — Supplementary Table 1 [file 41419_2020_2435_MOESM1_ESM.docx]

| **Gene list** | **Primer sequence** | |
| --- | --- | --- |
| **β-catenin** | F: gctttcagttgagctgacca | R: caagtccaagatcagcagtctc |
| **TWIST** | F: agctacgccttctcggtct | R: ccttctctggaaacaatgacatc |
| **Slug** | F: ccatgcctgtcataccacaa | R: acagtgatggggctgtatgc |
| **Snail** | F: aggatctccaggctcgaaag | R: tcggatgtgcatcttgagg |
| **Sox4** | F: agccggaggaggagatgt | R: ttctcgggtcatttcctagc |
| **SP1** | F: tttggcctcaaaacagaagc | R: ccaaacttcctgtggaagaga |
| **STAT3** | F: ctctgccggagaaacagg | R: ctgtcactgtagagctgatggag |
| **Bmi** | F: ccattgaattctttgaccagaa | R: ctgctgggcatcgtaagtatc |
| **CDH1** | F: ggtctgtcatggaaggtgct | R: gatggcggcattgtaggt |
| **c-Myc** | F: gctgcttagacgctggattt | R: taacgttgaggggcatcg |
| **Nanog** | F: atgcctcacacggagactgt | R: cagggctgtcctgaataagc |
| **Oct-4** | F: caatttgccaagctcctga | R: agatggtcgtttggctgaat |
| **SirT1** | F: gccagtggattcgctcttt | R: aatttcatcaccgaacagaagg |
| **Survivin** | F: caatttgccaagctcctga | R: agatggtcgtttggctgaat |
| **GAPDH** | F: gactaaccctgcgctcctg | R: gcccaatacgaccaaatcag |

**Supplementary Table 1:** Primer sequences used for RT-PCR assay
