# Supplementary figures and images for: CCL5 derived from tumor-associated macrophages promotes prostate cancer stem cells and metastasis via activating β-catenin/STAT3 signaling
Source: Cell Death Dis. 2020 Apr 16;11(4):234. doi: 10.1038/s41419-020-2435-y (PMC7162982; doi:10.1038/s41419-020-2435-y)

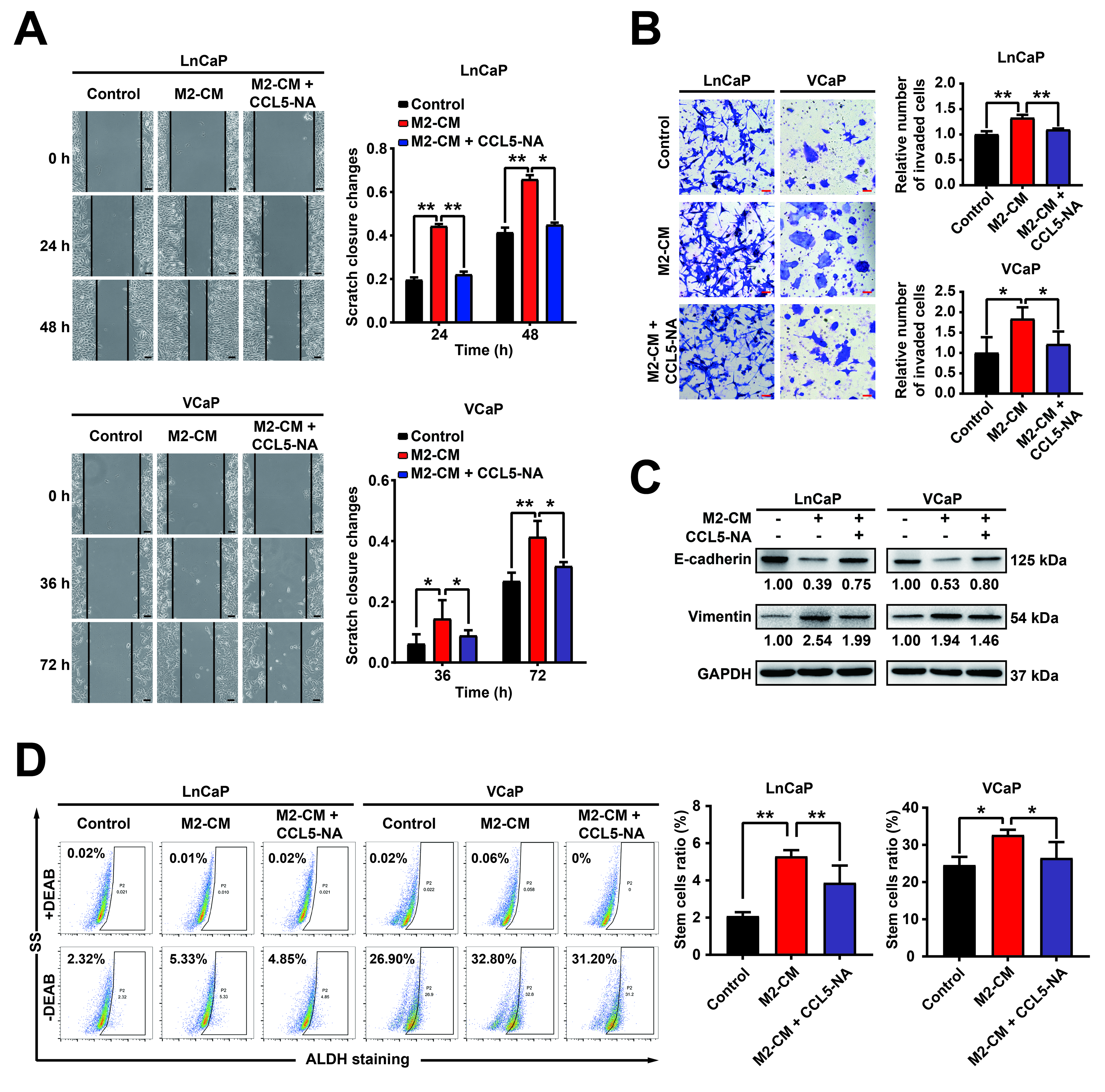

Supplement: Supplementary file 2 — Supplementary Figure 1 [file 41419_2020_2435_MOESM2_ESM.tif]
