# Supplementary material for: CCL5 derived from tumor-associated macrophages promotes prostate cancer stem cells and metastasis via activating β-catenin/STAT3 signaling
Source: Cell Death Dis. 2020 Apr 16;11(4):234. doi: 10.1038/s41419-020-2435-y (PMC7162982; doi:10.1038/s41419-020-2435-y)
Supplement: Supplementary file 3 — Supplementary Figure Legends [file 41419_2020_2435_MOESM3_ESM.docx]

**Supplementary Figure Legends**

**Supplementary Figure 1. TAMs-secreted CCL5 promoted the invasion and the PCSCs subpopulation in AR-positive LnCaP and VCaP cells.** **(A-C)** The CM of THP1-derived TAMs significantly promoted migration, invasion and EMT in AR-positive prostate cancer cell lines LnCaP and VCaP, while CCL5 NA could partly abrogate that. Scale bars represent 100 μm for wound healing assay images and 50 μm for transwell assay images. **(D)** The CM of THP1-derived TAMs significantly promoted the ALDH^+^ PCSCs subpopulation in LnCaP and VCaP cells, while CCL5 NA could partly abrogate that. All values are presented as the mean ± SD. n = 3, ^*^*p* < 0.05, ^**^*p* < 0.01.
